# Metropolitan Asylums Board

**Published:** 1897-07-03

**Authors:** 


### Metropolitan Asylums Board.

The Statistical Committee of the Metropolitan Asylums Board have just issued their annual report. It is somewhat different in arrangement from those of former years in that certain details are removed from the body of the report to a collective Medical Supplement, which seems likely to form a very useful and interesting feature. It is to be edited by two of the medical superintendents, who will be chosen by their colleagues. The editors for 1896 are Dr. Caiger and Dr. Goodall, and the subjects dealt with are the complications of scarlet fever and diphtheria, and a review of the post-scarlatinal diphtheria cases which arose during the year.

It is not without interest to note the frequency with which scarlet fever was complicated by other infectious diseases. Of these diphtheria was the most frequent, having occurred in 705 cases. Chicken-pox comes next with 486 cases, and measles follows with 167 cases. Rötheln occurred 73 times. Out of the 15,176 cases of scarlet fever treated, there were 1,162 cases in which simple albuminuria was met with, and 523 cases of nephritis.

In regard to these cases, it is somewhat important to observe that the great mass of them occurred at the acute hospitals, there having been only eight cases of nephritis at Gore Farm, and only 14 at the Northern Hospital. Clearly, then, they are not the result of premature removal to the convalescent hospitals.

The number of cases of otitis, viz., 2,290, seems rather large, and we would suggest as an interesting and most important field of study a comparative investigation into the treatment of this very serious complication. Among the 4,296 cases of diphtheria albuminuria appeared in 2,298, this number including all cases in which the faintest trace of albumen was detected at any time during the patients' residence in hospital. The cases of nephritis amounted to 24, or only 0·5 per cent. These numbers should be borne in mind in regard to the assertions sometimes made as to the effect of anti-toxin on the kidneys.

The number of cases in which paralysis complicated diphtheria was 883, or 20·5 per cent. of the whole; but this term includes all degrees of impairment of muscular power, such as a mere weakness of palate. On the other hand it does not include a simple loss of patellar reflex. The frequency of relapse is somewhat interesting, an actual return of exudation having been noted in 76 cases.

Turning to the body of the report, we find that during the year, 42,876 cases eligible for the hospitals of the Board were notified, and that of these 22,273, or 51·94 per cent., were actually admitted. The spot maps, which accompany the report, show that diphtheria especially prevailed in certain districts, particularly in the East-end, in Islington, Chelsea, Battersea, Newington, Camberwell, and Greenwich. They also show several aggregations of cases of typhoid fever, notably in St. Pancras, Islington, Shoreditch, and Poplar, which suggest the existence of some local sanitary defects favouring the distribution of the infection in those districts.

The ambulance service of the Board did an enormous amount of work during the year. Altogether 44,374 patients were removed; about half of these were primary removals to hospital, the rest being transferences of recovering patients to the convalescent hospitals, and back to London when well, and removals of other infected persons, among whom must be mentioned the 1,287 persons taken from the out-patient departments of general hospitals to their own homes at times when there were no vacant beds in the fever hospitals. Altogether the land ambulances ran 296,792 miles, and the steam boats 10,333 miles. These figures show the immense service done to London by the ambulances of the Asylums Board. In another year or two the Board hope to have nearly 6,000 beds for infectious diseases other than small-pox, and then their scheme will be considered complete.

Among the tables in the report is one giving the amount of illness which has occurred among the members of the staff at the various hospitals. There were 3,542 persons employed at the fever hospitals during the year, and of these 176, or 5·0 per cent., fell ill with fever or diphtheria, and three died. During the same period 220 persons were employed on the small-pox ships, of whom, however, none suffered from small-pox. From "other forms of illness," however, those on the ships suffered rather more than those on land. We would draw attention to the very different amount of illness which there seems to have been among the staffs of the different hospitals. Thus at the Eastern Hospital 136 persons were warded, and at the North-Eastern 148; while at the North-Western and Northern the numbers were only 36 and 51 respectively. The number employed is approximately the same at each place. No doubt this matter has received the consideration of the managers, but it certainly is one which requires investigation. We regret to see that there were 12 cases of typhoid fever; two of these, however, occurred at hospitals where that disease is not admitted and were doubtless accidental. Taken altogether, the report is a very satisfactory record of an immense amount of good work which is being quietly done in our midst, greatly to the benefit of the community.

